# Cultural Understanding of Wounds, Buruli Ulcers and Their Management at the Obom Sub-district of the Ga South Municipality of the Greater Accra Region of Ghana

**DOI:** 10.1371/journal.pntd.0004825

**Published:** 2016-07-20

**Authors:** Eric Koka, Dorothy Yeboah-Manu, Daniel Okyere, Philip Baba Adongo, Collins K. Ahorlu

**Affiliations:** 1 Noguchi Memorial Institute for Medical Research, University of Ghana, Legon, Ghana; 2 School of Public Health University of Ghana, Legon, Ghana; 3 University of Cape Coast, Cape Coast, Ghana; University of Tennessee, UNITED STATES

## Abstract

**Background:**

This study was conducted with the aim to understand some of the cultural belief systems in the management of wounds and patients practices that could contaminate wounds at the Obom sub-district of the Ga South Municipality of Ghana.

**Methods:**

This was an ethnographic study using in-depth interviews, Focus Group Discussions and participant observation techniques for data collection. Observations were done on Buruli ulcer patients to document how they integrate local and modern wound management practices in the day-to-day handling of their wounds. Content analysis was done after the data were subjected to thematic coding and representative narratives selected for presentation.

**Results:**

It was usually believed that wounds were caused by charms or spirits and, therefore, required the attention of a native healer. In instances where some patients’ wounds were dressed in the hospital by clinicians whose condition/age/sex contradict the belief of the patient, the affected often redress the wounds later at home. Some of the materials often used for such wound dressing include urine and concoctions made of charcoal and gunpowder with the belief of driving out evil spirits from the wounds.

**Conclusion:**

Clinicians must therefore be aware of these cultural beliefs and take them into consideration when managing Buruli ulcer wounds to prevent redressing at home after clinical treatment. This may go a long way to reduce secondary infections that have been observed in Buruli ulcer wounds.

## Author Summary

The study revealed a number of cultural practices and beliefs which influenced patients' wound care and health seeking behaviour. These included the beliefs that prohibit certain category of people such as pregnant women, lactating mothers and women who menstruate from dressing wounds. Respondents believed that some wounds were caused by charms or spirits and, therefore, required the attention of a traditional healer. In instances where patients’ wounds were dressed in the hospital by clinicians and the patients observed that the condition, age or sex of the clinician contradict their belief, they often redressed the wounds later at home for fear of the wound not healing. Some of the materials often used for such wound dressing include urine and concoctions made of charcoal and gunpowder with the belief of driving out evil spirits from the wounds. These practices may cause secondary infection of wounds considering the conditions under which the mixtures (concoctions) are prepared. It may require collaborative efforts of clinicians, public health promoters and the affected communities to find a common ground to manage Buruli ulcer wounds in a mutually acceptable way to aid healing.

## Introduction

Buruli ulcer, caused by the environmental pathogen *Mycobacterium ulcerans* is an important cause of morbidity especially in West Africa, where it is ranked the second most important mycobacterial disease [1]. The mode of transmission of the pathogen is still elusive and therefore control relies mainly on case detection and treatment, which comprises of eight weeks of Streptomycin and Rifampicin followed by surgery to correct deformities when needed [1]. The success of this treatment modality depends very much on detecting cases early. However due to socio-cultural and economic factors, most cases are detected very late with large wounds with massive cell destruction by the cytopathic toxin, mycolactone [1]. Therefore wound management is an important component in buruli ulcer case management. At the same time, Microbiological research has revealed numerous secondary infections among buruli ulcer patients at the endemic districts of Ga West and South [1]. As attempts are being made by microbiologists to establish the cause of these secondary infections among the patients, there is the need for social science research to complement what microbiologists are doing to determine the source of secondary infections. In addition, there is a need for an in-depth ethnographic study to understand the socio-cultural beliefs and practices associated with management of wounds in buruli ulcer endemic communities to aid clinical practice. This study was conducted with three main objectives; (1) to understand cultural beliefs and practices associated with buruli ulcer wound care, (2) to determine the relationship between these beliefs and practices on one hand and wound contamination on the other hand at the Obom sub-district of the Ga South Municipality in Ghana, and (3) to determine and describe whether patients’ perceptions of wound management at health facilities meet their expectations in terms of culturally acceptable wound handling in study communities.

This study is important because, the effectiveness of therapeutic relationships between wound care clinicians and patients is highly dependent on the understanding of what is culturally appropriate in wound management. As reported by Wessels [2], culture is a vital aspect of wound care planning and management in most part of Africa.

### Ethical consent

The Institutional Review Board of the Noguchi Memorial Institute for Medical Research, University of Ghana reviewed the study. The following ethical considerations were followed:

Ethical principles of anonymity, confidentiality, and rights of withdrawal were ensured among participants (buruli ulcer coordinators and patients, and all other respondents). Research participants were informed about the objectives, methods and what was expected of them by clarifying their roles in the study to them. It was made clear to participants that their participation in the study was voluntary and refusal to take part would not affect their access to services offered by the health facility. No form of inducement was used to entice participants to partake in the study. However, refreshment and transportation was provided after interviews. To help protect the identity of patients and prevent questioning by community members, both the questionnaire administration and individual interviews were held within an environment devoid of many people, which were largely chosen by respondents. To ensure participants’ right, an informed consent (both oral and written) was obtained from them before conducting the interview.

## Methods

### The study area

The study took place in the Obom sub-district of the Ga South Municipality. The Ga South Municipality was carved from the then Ga West District in November 2007. The Municipality was established by Legislative instrument 1987 in 2007 with the capital at Mallam. The Ga South (Weija) Municipal Area lies within Latitude 5 degree 48’ North and within Longitudes 0o 8’ East and 0o 3’ West. It has total land coverage of approximately 517.2 sq. km. It shares boundaries with Accra Metropolitan Area to the South-East, Ga West to the East, Akwapim South to the North- East, West Akim to the North, Awutu Senya to the West, Gomoa to the South-West and the Gulf of Guinea to the South [3].

According to the extract from the 2010 National Population and Housing Census, the total population of the district is approximately 485,643 made up of 248,085 (51.1%) females. The high population size is due to the Municipality’s closeness to the capital City Accra, making it home for many workers. According to the 2010 census, there are about 362 communities spread in the urban, peri-urban and rural areas of the Municipality. The coastal and the central portion of the Municipality have very dense population while the communities in the northern section are sparsely populated and scattered [3].

Obom, a sub-district in the Ga-South Municipality is located 15 kilometres to the north-east of Amasaman the district capital of Ga West Municipality. The Eastern part of the sub-district consists of low hills, interspersed with plains in the central parts. The river Densu, the largest water body in the district, runs through the sub-district. Other water bodies, which are tributaries of the Densu, are Adeiso, Honi and Ponpon rivers. There are also small ponds and seasonal streams. In addition, numerous surface water bodies have sprung up in the wake of extensive sand-winning activities to supply the building industry in the sub-district and the neighbouring Accra metropolis with sand. These water bodies are significant for economic activities such as fishing and farming as well as disease causation. Water—related diseases such as Buruli ulcer, schistosomiasis and malaria are endemic in the sub-district [4].

Apart from the two main health facilities (one in Obom, the sub-district capital and the other in Amasaman, the district capital of Ga West Municipality) that are accessible to residents in the sub-district, there are private clinics and maternity homes in the sub-district, some of which are at Mayra, KojoAshong, Domeabra, Oduman and Jei-Krodua. These facilities complement the efforts of the sub-district public health delivery, which could not reach majority of the people due to poor access and coverage. There are other decentralised health facilities (CHPS compounds) at Ashalagya, Balagono, Hobor and Kofikwei providing primary health care services to the populations that they serve. Owing to the poor condition of roads, the scarcity of means of transport and the fact that most communities are quite far from health facilities, access to health care is a major problem in the sub-district. The majority therefore utilise home treatment either homemade herbal treatment or over the counter medications usually bought from shops and itinerary vendors to manage ailments as a first line of action [4].

### Study design

This was an ethnographic study using in-depth interviews, focus group discussions and participant observation techniques for data collection. Interviews and focus group discussions (FGD) were conducted with selected community elders, traditional healers, buruli ulcer patients and some patient caretakers in selected communities. In all, fifty five in-depth interviews and three focus group discussions were held in the study area. There were eight participants in each FGD session. Also, observations were done on buruli ulcer patients to document how they integrate local and modern wound management practices in the day to day handling of their wounds. Content analysis was done after the data were subjected to thematic coding and representative narrative selected for presentation.

### Data collection procedure

A recruitment strategy (plan for identifying and enrolling people to participate in the research study) was used. The strategy specified the criteria for screening potential participants, the number of people to be recruited, the location and the approach. In recruiting people for the FGDs the inclusion criteria were elderly male and female above age 55 years living in the communities that were sampled. Male and female above age 55 were considered elderly and appropriate to provide the necessary information on culture of wounds in this study based on consultations with the community chiefs and elders. With the aid of community volunteers and a disease control officer, both of whom had fair knowledge of the social setting of communities, a Ga, Ewe and Akan community elder, the three dominant ethnic groups living in the study area, were first approached by introducing the research team and the aims and objectives of the study to them, then asking them to kindly participate in the study. It was further explained that participation was voluntary and that one was at liberty to withdraw from the discussions anytime one deemed it necessary. However a promise to keep the discussions as confidential as possible was also made. When these elders consented to participate, we got the other members through a kind of snowballing in which the participant with whom the contact had been made and agreed used their social networks to refer the team to other elders who could potentially participate in the study.

The same strategy was employed for selecting respondents for in-depth interviews with selected elderly community members. Convenient sampling was used to select buruli ulcer patients and their corresponding caretakers who came to the clinic for treatment or went to traditional healers for treatment. Traditional healers were identified using their association president.

### In-depth interviews

In-depth interviews were conducted with selected buruli ulcer patients on treatment at the Obom health center, caretakers of patients, selected elderly community members, and traditional healers to solicit information on buruli ulcer treatment, personal/community beliefs about wound care and perceptions of wound care at the biomedical health facilities.

Fifty five in-depth interviews were conducted (five buruli ulcer patients, five caretakers of patients); 30 elderly community members (10 each of the dominant ethnic groups in the study area) (Ewe, Ga and Akan) and five traditional healers. Also 10 key informant interviews were conducted with health care providers to appreciate the challenges facing the health system when it comes to Buruli ulcer control efforts. The key informant interviews were all done in English. With exception of the traditional healers who were all men, women were equally represented.

Key-informants in this study were health care managers and providers comprising of the Director of the National Buruli ulcer control programme, the in-charge of the Obom health centre and nurses in the wound dressing room, the District Director of Health Service. Key-informant interviews allowed for the inclusion of providers’ perspective on wound care at the health facility. They also drew attention to the manner in which policies were applied and the challenges faced during day-to-day operations. Thus, key-informant interviews were critical in bringing biomedical context into the study.

### Focus group discussions

In other to generate interactive consensus building processes in the communities, three focus group discussions (FGDs) were held (one each with the dominant ethnic groups in the study area) (Ga, Ewe and Akan) respectively. Just like the in-depth interviews, FGDs were done to solicit information on buruli ulcer treatment, personal/community beliefs about wound care and perceptions on wound care at the biomedical health facilities.

### Observation

This method employed an interpretive paradigm which aimed at understanding the dynamics of the socio-cultural system as well as how communities interpret their world [5]. With this method, more time was spent in the communities in order to observe the daily activities of the people. The focus of the observation was on the daily management of wounds (BU or suspected BU) in selected households. The households selected were houses where buruli ulcer patients who started treatment at the health facility but defaulted or dropped out of treatment were residing. Additionally, community members who were identified as having wounds but decided to manage it at home were purposively visited. We also observed traditional healers’ ways of managing wounds of their clients. The observations were complemented by questions to clarify and understand individual actions taken in the process of wound care/management.

### Data management and analysis

Phenomenological analysis was performed on the qualitative data. Qualitative data from in-depth interviews and focus group discussions were categorised in a format that allows for manual coding by interview item for content analysis to be done. Data was analysed to clarify aspects of cultural beliefs of wound care, experiences of traditional treatment of wounds and treatment seeking behaviour of patients. Qualitative variables of interest were categorised and selected into common themes for presentation. This allowed the performance of phenomenological analysis on relevant coded segments to select representative narratives for presentation to complement the quantitative data.

## Results

Socio-cultural beliefs were found to influence the perceptions of respondents on their categorisation of wounds in a buruli ulcer endemic district. Many of the respondents believed that some wounds were caused by witches, evil spirits and ancestral spirits.

### General wounds and buruli ulcers

From the medical perspective, a general wound is a type of injury in which the skin is torn, cut, or punctured (an open wound), or where blunt force trauma causes a contusion (a closed wound). However, we found that the biomedical care providers in our study also classified wounds into four main categories as follow:

- Clean wound, a wound kept under sterile condition where there is no organism present in the wound and the wound is likely to heal without complications.
- Contaminated wound, where the wound is as a result of accidental injury with pathogenic organisms and foreign bodies in the wound.
- Infected wound, where the wound has pathogenic organisms present and multiplying showing clinical signs of infection, where it looks yellow, oozing pus, causing pain and redness.
- Colonized wound, where the wound is a chronic one and there are a number of organisms present and very difficult to heal.

Further categorisations of wounds are acute and chronic wounds. Acute wounds are wounds that heal within a short period of time while chronic wounds are difficult to heal or last for a very long period of time. However, the following narratives by a biomedical care provider provided clear distinctions between other wounds and buruli ulcers wounds;

*‘‘…*..*Buruli ulcer is a chronic skin and soft tissue infection caused by the Mycobacterium ulcerans*. *Unlike other wounds*, *buruli ulcer often starts as a painless swelling (nodule)*. *It can also initially present as a large painless area of plaque or a diffuse painless swelling of the legs*, *arms or face (oedema)*. *Local immunosuppressive properties of the mycolactone toxin enable the disease to progress with no pain and fever*. *Without treatment or sometimes during antibiotics treatment*, *the nodule*, *plaque or oedema will ulcerate within 4 weeks with the classical*, *undermined borders with the necrotic tissue (cotton wool-like) at the edges*. *Occasionally*, *bone is affected causing gross deformities…*..*”* (Key informant interview, male medical assistant).

He further explained that;

*‘‘…*..*For the natural wounds*, *the management/dressing depends on the type*, *cause*, *and depth of the wound as well as whether or not other structures beyond the skin (dermis) are involved*. *Minor wounds*, *like bruises*, *will heal on their own*, *with skin discoloration usually disappearing in 1–2 weeks*. *Abrasions*, *which are wounds with intact skin (non-penetration through dermis to subcutaneous fat); usually require no active treatment except keeping the area clean*, *initially with soap and water…*.*”* (Key informant interviews, a male medical assistant).

Residents in BU endemic communities however, categorised wounds into two types—the normal wounds and the abnormal wounds. The normal wounds among the Ga it is called *fla*, the Ewe called it *Abi* and the Akan called it *Akuro*. These are wounds that were as a result of cuts, falls, boils, bites and accidents of all kinds. They heal very fast, usually within three months. The abnormal wounds, known locally as *aboabone* (Ga), *akuro bone* (Akan) or *abivordi* (Ewe) are those that may start as normal wounds but due to other interventions, may take a very long time to heal or may not heal at all. Abnormal wounds (could also be caused by supernatural forces notably witches, wizards, ancestral spirits, charms/sorcery/ Juju and the gods of the community. Thus, community members believed that wounds can be caused by natural factors, supernatural factors or both. They also believed that wounds are living beings that could sleep and wake up and this belief actually influences when a wound should be dressed and not to be exposed for all to see. They also believe that wounds must be dressed by elderly people but not young people. The people also believe that chronic wounds must be managed by traditional healers and not to be taken to the biomedical health facilities.

According to some of the traditional healers visited, violations of some beliefs and prohibitions mentioned above could lead to serious consequences including the non-healing of wounds, barrenness, and even death. For some lucky victims, pacification rites in the form of libation are performed by linguist and fetish priest in the communities to avert the calamity or the punishment.

### Community perceptions on wounds, buruli ulcers and their management

**Fig 1:**
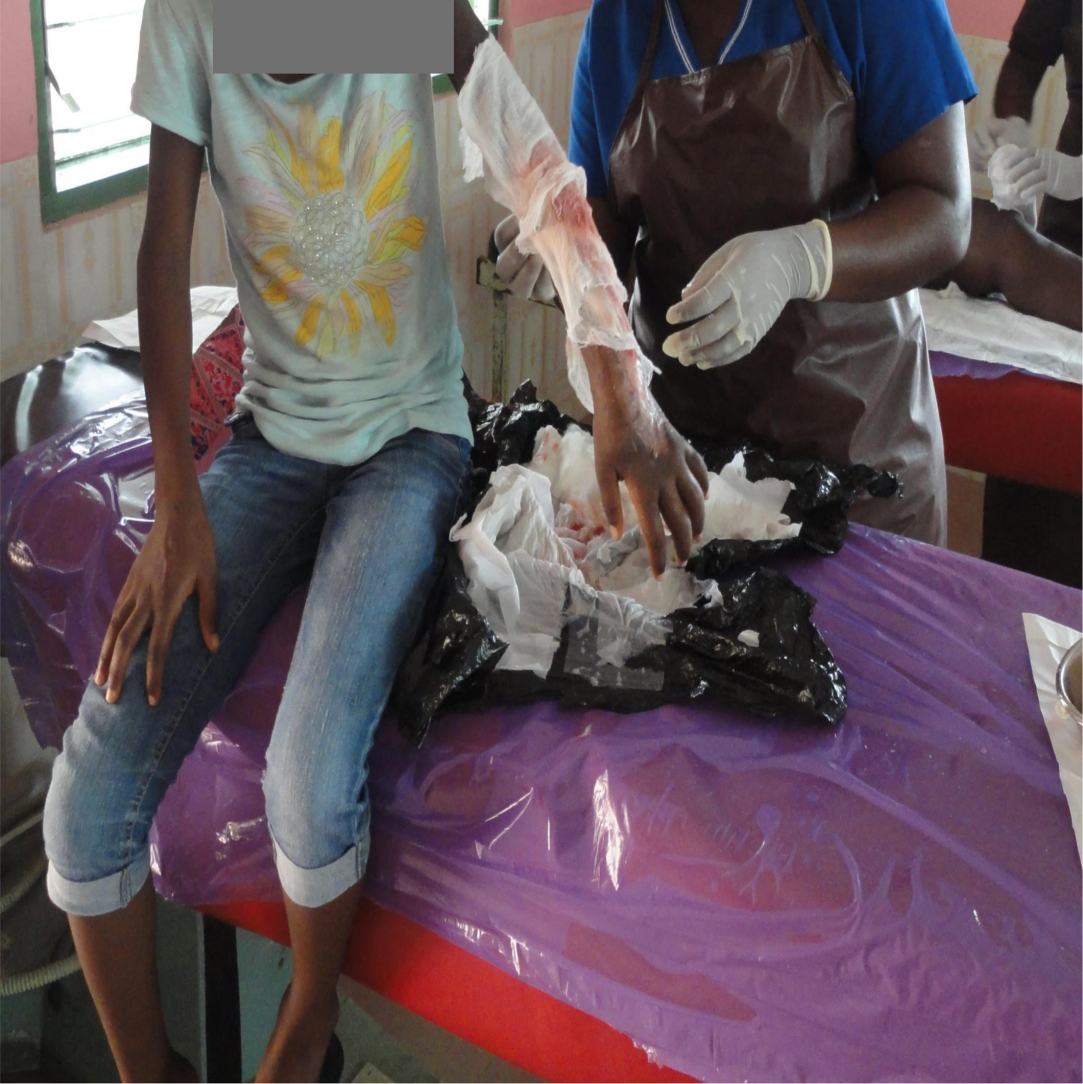
Dressing of buruli ulcer wound at a health centre.

The treatment of Buruli ulcer can be straight forward and less costly if the disease is detected early without complications. However, treatment becomes more costly if found in the advanced stage (Fig 1). The basic treatment consists of antibiotics taken daily, both orally and injection for 56 days, coupled with wound care/management. One of the following combinations may be used: a combination of rifampicin (10 mg/kg once daily) and streptomycin (15mg/kg once daily); or a combination of rifampicin (10 mg/kg once daily) and clarithromycin (7.5 mg/kg twice daily) (Observations). Also, other interventions such as limited physiotherapy services are provided to minimise or prevent disabilities (Fig 1).

Community members believed that normal wounds could be managed at home or at the biomedical health facilities while abnormal ones should be managed by the traditional healers/spiritualists/herbalists. To buttress this position, one community member said;

*‘‘…For normal wounds*, *we mix antibiotics such as ampicillin and amoxicillin with palm kernel oil and applied mixture on the wound twice daily*. *Herbal mixtures are also applied on the wound by some people…*..*”*(A female community member, field notes).

**Fig 2:**
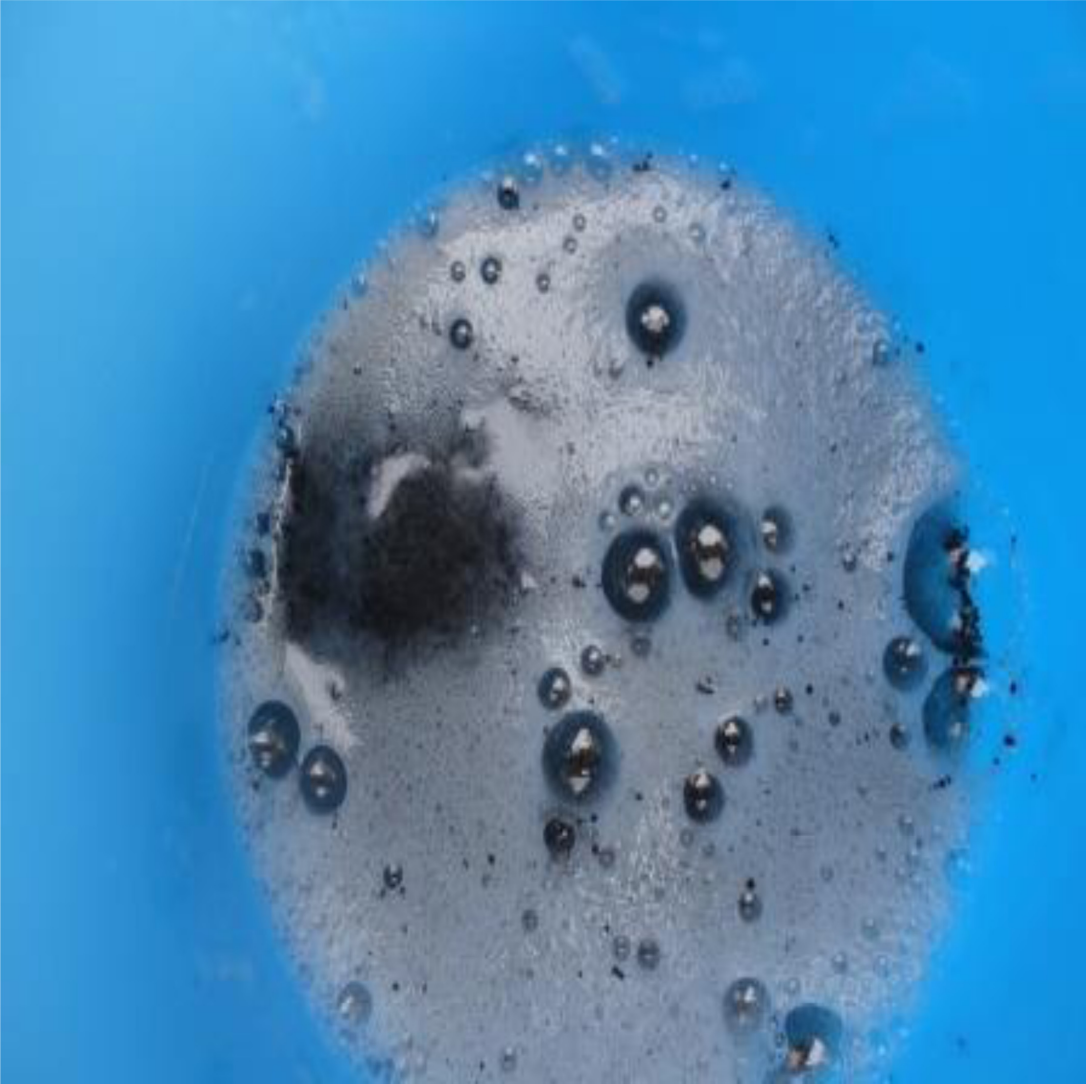
Concoction of charcoal, gunpowder and palm wine.

**Fig 3:**
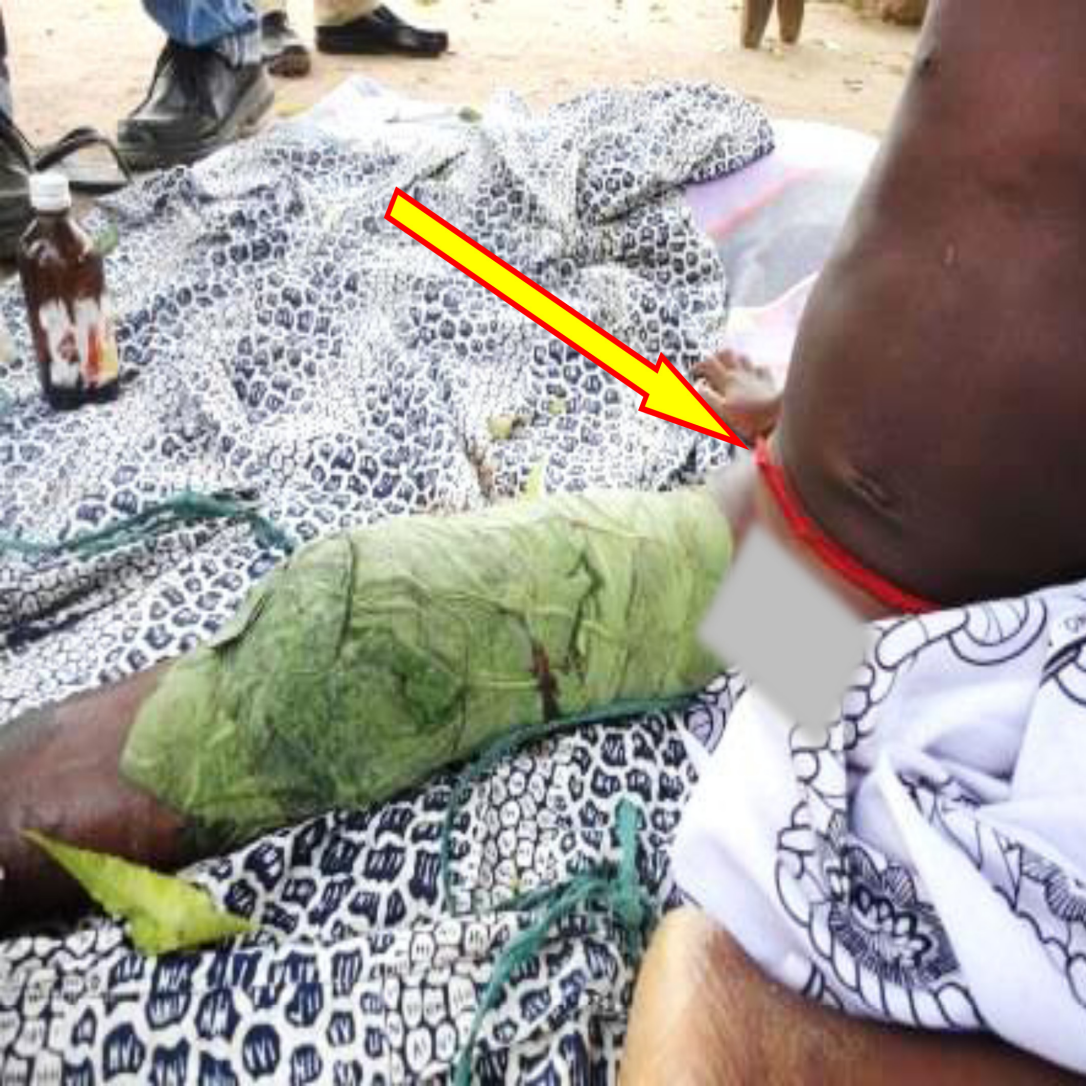
A Talisman around the waist to ward of spiritual attacks protection. The wound was covered with ‘Kontomire’ (Cocoyam Leaves) to aid healing.

Some community members believed that buruli ulcers wound by their nature, fall into the category of abnormal wounds and so need to be managed traditionally with spiritual backing for fast healing. The process of managing buruli ulcer wounds by traditional healers involves, first casting out the causal spirits before applying various herbs on the wound to aid healing. The processes of managing buruli ulcer by a traditional healer were observed in one study community. Traditional healers as follows: evil spirits are driven out of the wound by making the patient to drink concoctions made up of a mixture of palm wine and gun powder (Fig 2). For long lasting protection from the causal spirit, the patient is made to wear a red talisman around the waist (Fig 3). The final stage of the treatment process involves putting herbs such as cocoyam leaves (kontomire) among other herbal preparations on the wound to aid healing (Fig 3).

**Fig 4:**
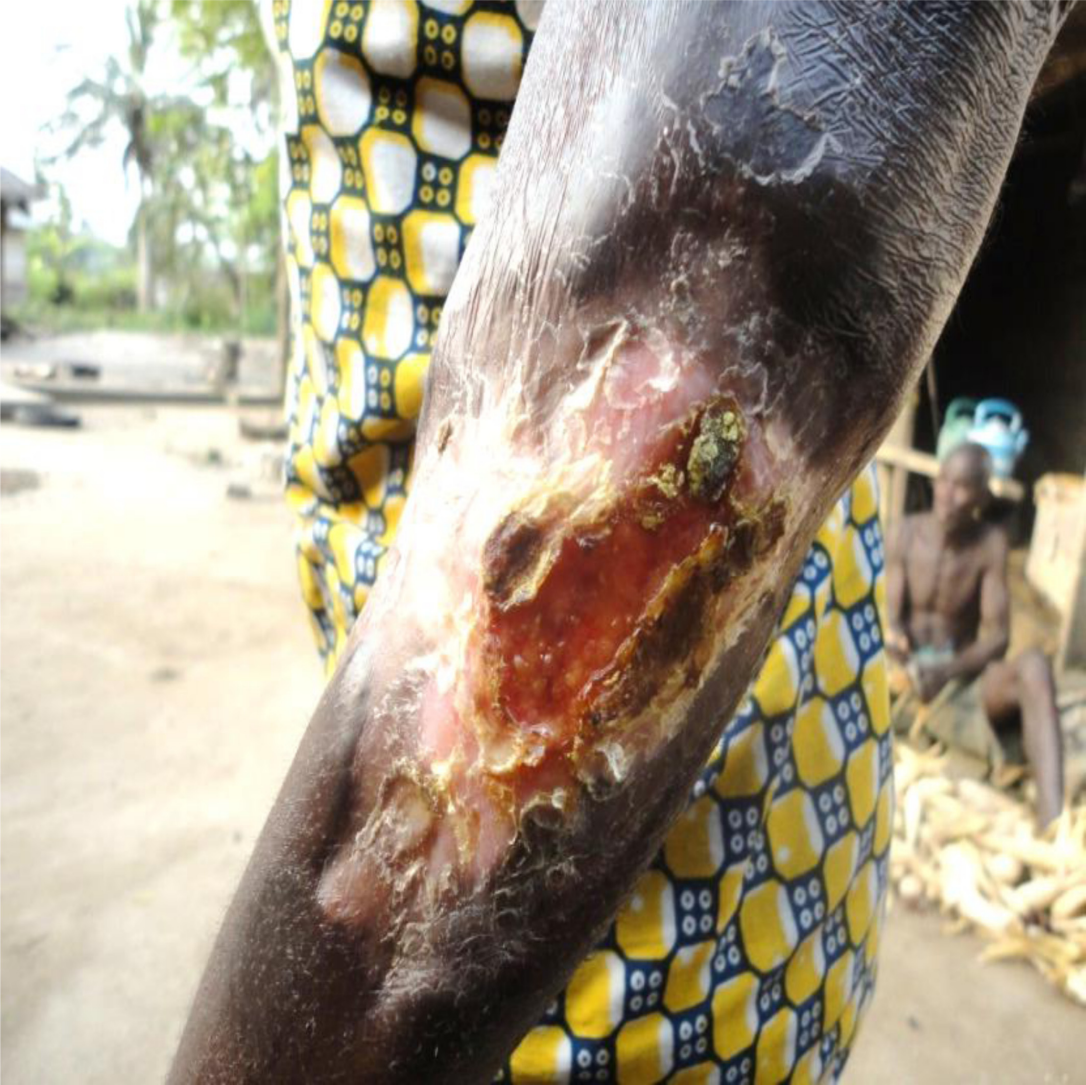
Dryness of skin indicating sign of a healing wound.

Unlike the biomedical perspective, where moist wounds are considered to be in good conditions, community members perceived dry wounds as those that are showing signs of healing (Fig 4). This belief was confirmed by a buruli ulcer patient who dropped out of biomedical treatment and went to a traditional healer. In her narration, she said;

*…*..*When I was receiving treatment in the clinic*, *my wound was not healing because it was always wet or moist……but since I came here*, *it was becoming dry showing signs of healing…” (*A 21year old Buruli ulcer patient, Field note).

This assertion was confirmed by the traditional healer who was managing the wound when he said:

*‘‘……*..*When she came to me for treatment*, *I saw that the wound was wet showing how bad it was but after some weeks I realised that it has become dry*, *which is an indication that the wound is healing…”* (A 73 year old traditional healer, observation)

Respondents were of the opinion that, depending on the causes and classifications of wound, some could only be managed by traditional healers, spiritualists or herbalists while others could be managed effectively by biomedical health workers. This position was reflected in a statement by a 76 year old female respondent during a focus group discussion session when she said;

*‘‘…*.*There are two types of wounds*…*Clean wounds (Ekro)… they healed within three months and Chronic wounds (Kisikro)*…*they take long time to heal … years and some can be for life…these are the kisikro (ulcers)…*.*Clean wounds are ordinary wounds which come about as a result a cut*, *burn*, *fall*, *and any other occurrence that inflicts injury on an individual…*..*I know herbs that are used for the treatment of clean wounds…We use “acheampong”(Chromolaena Odorata) herb among others for the treatment… the herbs are washed*, *grounded and smeared on the wound…*.*For the clean wounds*, *you can go for the herbs at any time and anyone can go for it*..*…Some of the herbs are boiled and used to wash the wounds…Several herbs can be combined to wash the wounds…”* (An Akan female participant, focus group discussion).

The chronic wound (Kisikro) was also described by a 70 year old female during an in-depth interview session as follows:

*‘‘…*.*The chronic wounds (Kisikro) are wounds that last for over three months…*..*when wounds (Ekro) failed to heal within three months*, *they are called chronic wounds (kisikro)… at their early stage*, *they are treated with rotten plantain buds*……*it is at this stage that people attribute its cause to the spirits and witchcrafts…*.*People then begin to speculate that witches are cutting/chopping meat on the affected part…”* (An Akan female respondent, in-depth interviews).

To buttress this belief, a 67 year old male participant had this to say:

*‘‘…We*, *the Ga people believe that some wounds are caused by witches*, *evil spirit*, *and ancestors… wounds that do not heal after three months…*. *People with such wounds can only be treated by the traditional healer-herbalist or spiritualist…*.*”* (A Ga male participant, focus group discussion).

To show that the belief regarding wound causation is similar in Southern Ghana, we present what a 65 year old woman said during an in-depth interview session:

*‘‘…*.*I have been managing wounds but not all wounds……I manage ordinary wounds but not those that are perceived to have been caused by witchcraft…*. *Spirit/bewitched wounds do not heal fast…*.*they last more than 3 months…*…*”* (A 65 year old female Ewe respondent, in-depth interview).

Local beliefs about the causation of wounds affect the treatment practices of traditional healers in treating wounds in general and buruli ulcer wounds in particular. This has been expressed by a traditional healer during an in-depth interview session thus:

*‘‘…*.*When the case is brought*, *I take it into prayers and get revelations about its causes until the following day and if it is being caused by supernatural forces …I tell the patient and the family about the items needed in order to cure the disease…*.*The items are used to pacify the causal agents (witches) who visited the disease on the patient…because it is a spiritual cause*, *once I identify the spirits (witches)*,..... *This spirits come in the night to take the patient away… After given them whatever they demanded …they (witches) direct me to where the things are to be sent…I then go with the patient and the family to the place mostly at 12 midnight when everyone is asleep…”*(A traditional healer, in-depth interview).

Asked why the witches inflict wounds on victims, this was what the respondents said:

*‘‘…At times*, *it is just a sheer hatred for no apparent reason…at times too*, *rivals also punish each other through bewitching and people who quarrel with others or insult others are punished with the wounds… They derive satisfaction and joy from inflicting pain on such people…”* (A traditional healer, in-depth interview).

Chronic wounds are perceived and treat them differently in line with the belief of the people. This belief was stated by a discussant in a focus group discussion session as captured in the following narratives:

*‘‘…We (the Akans) also believe that some wounds are caused by spirits and witchcrafts…once it is believed that the wound is caused by spirits or witchcrafts*, *then you have to go and see the fetish priest or herbalist/spiritualist/traditional healer…*.*For the chronic wounds (ulcers)*, *some people use fermented urine to clean the wounds… the fermented urine serves as alcohol use to clean wounds at the biomedical health facilities…”* (A 69 year old male Akan respondent, focus group discussion).

Despite the local perceptions that wounds are caused by spirits or witches, some community members including buruli ulcer patients are beginning to doubt these positions and this may be mainly due to the community outreach education programme being implemented in the study sub-district. This was reflected in a statement made by a 48 year old male Buruli ulcer patient during an in-depth interview when he said:

*‘‘…*.*There is a belief in my community that some chronic wounds are caused by witches and other spirits… Initially*, *I believed that my wound was caused by witches but since I started coming for treatment at the clinic I do not believe that anymore…*.*because I now know it is Buruli ulcer and is caused by bacteria instead of witches… We are being educated at the clinic and in our community…” (*A male buruli ulcer patient, in-depth interview).

Again, this was what a 72 year old female had to say about her perception of chronic wounds and how it has changed overtime:

*‘‘…*.*I used to believe that all persons with chronic wounds were witches or their conditions were caused by witches and wizards…*.*My belief really had a serious negative influence on my relationship with people with such conditions…*.*I gossiped about them and called them names…*.*Fortunately or unfortunately*, *my own daughter had a wound that failed to heal within 3 months…*. *We visited almost every traditional healer in and out of my community but to no avail…I became worried because people might start pointing accusing fingers at me as being the cause of my daughter’s condition as I did to others…Luckily for us*, *we were about visiting another traditional healer when a friend who had suffered from a similar condition directed us to the Obom health centre…*.*At the clinic*, *my daughter was diagnosed of ‘‘Odontihela” (buruli ulcer)*. *After the daily injections*, *the wound healed completely and my daughter became healthy again…*..*In fact*, *this changed my earlier belief about chronic wounds…”* (A 72 year old female focus group discussion participant).

### Cultural beliefs on who should dress wounds

The socio-cultural beliefs and practices associated with wound care in the study district also had ramifications for patients and their caregivers. Almost all the respondents alluded to the fact that wound care was delicate, complex and mysterious and so its management is restricted to particular categories of people. Respondents maintained that there were people who are not fit to go near someone with a wound let alone to dress it. It came to light from responses that; women who are breastfeeding, women who are in their menstrual period, pregnant women, promiscuous young women and people with ‘evil eyes are not supposed to dress wound. It is also belief that more than one person (multiple hands) should not dress a wound. The reasons behind this believe is that when these categories of persons dress your wound, it will not heal fast or heal at all.

According to a 68 year old woman shared her personal experience in the following narratives:

*…I had an experience where my own son was circumcised and I was managing the wound for him…*.*it was not healing till my mother took over and the wound got healed after a short period…*. *I was later told that it was because I was breastfeeding at the time that was why it was not healing… This made me to believe what our forefathers said…* (A female participant, focus group discussion).

Asked who was therefore qualified to take care of wounds, this was her response:

*‘‘…*.*It is better for a very old woman*, *who has stopped giving birth*, *having sex and does not breast feed to dress wounds…*.*When such a person is dressing your wound*, *it heals fast because they are considered as very clean*, *pure and experienced to handle the wound…” (*A female participant, focus group discussion).

On the other hand, most of the participants believed that people with wounds were not supposed to engage in the following acts if they want their wounds to heal fast: They must not have sex until the wound gets healed; They must not dress the wound in the afternoon; They must not dress the wound outside a room; They must not expose the wound for all to see as some people have evil eyes which could affect the wound. This position was confirmed in the following narratives:

*…We (the Ga people) believe that a wound does not heal fast when a lactating woman is dressing it… It was also believed as a taboo for a fetish priest to see wounds…*.*If a fetish priest sees a wound it does not heal fast…*. *It was because such people had charms/juju that prohibits them from seeing wounds*.....*However*, *if a fetish priest sees a wound and decides to have mercy on such a person*, *he/she would ask you to come for treatment…*. *By so doing*, *he/she removes the spirit that would prevent the wound from healing from the wound… Most of the time*, *they use herbs or spit in the wound with some incantations…* (A Ga male, In-depth Interview).

Asked why people had to hide their wounds, their responses were captured in the following narratives:

*‘‘…Most people hide their wounds to prevent evil/bad eyes from seeing the wound…*. *Again that was why some people also did not want to bring their wound to the hospital because they did not want any evil eye to see the wound…*. *However*, *they also believed that people whose wounds last for several years were witches/wizards themselves…*. *They were believed to be using the wound as eating from it or use where the wound is as a chopping board…*. *Witches also inflict chronic wounds on other people either by eating from it or use it as chopping board in the spirit realm…”* (A male participants, focus group discussion).

In expressing the same sentiment about who is to dress wounds at the clinic, this was what a 64 year old female patient had to say:

*‘‘…Nurses dress my wound at the clinic and these nurses are male and females…*. *I am happy with the way some take care of me but some do not take good care of me at all…”*(In-depth interview, 64 year old female patient).

Asked whether she had ever been attended to by a pregnant nurse since she started coming for wound dressing, she replied:

*‘‘…*..*Yes there was a day that a pregnant woman (nurse) dressed my wound and I protested because of the belief I have that the wound would not heal fast or at all…*. *Some of us patients did not like that at all…*. *So when the in-charge heard it he talked to us and assured us that the pregnant nurse would not dress our wounds again…” (In-depth Interview*, *a* 64 year *old female respondent)*

In stating her state of health and how she was being treated by the nurses at the clinic, this was what the respondent said:

*‘‘…The condition in terms of improvement is on and off…*. *There are times when I saw significant improvement and at times it became worst…*.*I think some of the nurses do not talk to us well and are not patient with us…*.. *They should be advised to treat us with respect and like their mothers and fathers…*.. *Some of them shout on us even when we are in pain…” (*In-depth interview, *a* 64 year old female respondent)

Findings revealed a number of cultural practices and beliefs which significantly affected patients' wound care and help seeking behaviour. These included cultural beliefs that prohibit certain category of people such as pregnant women, lactating mothers and women who menstruate from dressing wounds. Respondents believed that some wounds were caused by charms or spirits and, therefore, required the attention of traditional healers. In instances where patients’ wounds were dressed in the hospital by clinicians and the patients observed that the condition, age or sex of the clinician contradict their belief, the affected often redressed the wounds later at home for fear of the wound not healing. Some of the materials often used for such wound dressing include urine and concoctions made of charcoal and gun powder with the belief of driving out evil spirits from the wounds. These practices may cause secondary infection of wounds considering the conditions under which the mixtures (concoctions) are prepared.

### Other factors that influence treatment seeking behaviour

The kind of relationship that exists between patients and care providers has a great influence on their treatment seeking behaviour and adherence to treatment. The relationship has psychological effects on patients’ healing process. It is therefore vital to respect and take into considerations the beliefs and practices of patients as much as possible so as to avoid conflict with the biomedical treatment. We tried to understand how patients perceived biomedical health practitioners who handle their wounds during treatment at health facilities and the following narratives explained their positions:

*‘‘…We (Akans) have the belief that government health workers like nurses and doctors are exempted from some of the taboos of wound care…*.*However; some nurses too have good and bad eyes…”*(In-depth interview, *a* 70 year old female respondent).

Asked how she saw the services being provided by the nurses, this was what she said:

*‘‘…Hmmmm*!!! *My son (*referring to the interviewer) *Without being apologetic*, *some of the nurses at the clinic do not deserve to be nurses because they do not treat patients well at all…*.*Such people do not work with a clean conscience; hence your condition will never improve nor heal…*..*So as for me*, *when I get to the clinic and ask of the in-charge called ‘chief’ and they say he is not there*, *I do not allow anyone to attend to me…”(*In-depth interview, *a* 70 year old female respondent).

In suggesting how nurses are to treat patients with wounds at health facilities, a 66 year old male respondent said:

*‘‘…*..*People who have wounds need to be pampered for the wound to heal fast…*.*So nurses need to pamper patients so that they do not dropout of treatment…*. *There are nurses who do not take good care of wounds when they are dressing them… They have no time for the patient so such patients’ wounds do not heal fast or they stop coming for treatment at the clinic…” (A* 66 year old male, focus group discussion).

While some of the patients interviewed maintained that they did not mind when pregnant nurses dressed their wounds for them at the clinic, others were not comfortable with that. These positions were represented in the following narratives:

*‘‘…In the clinic*, *there are young nurses who dress my wound for me…*. *I am happy with the way some of them treat/dress the wound but some are not polite at all…*. *There was a pregnant nurse who dressed my wound…*. *But since she is a government worker I am not worried…*. *However*, *some of the patients especially the old women were not happy with that…*.. *They were not happy because of the belief that pregnant women are not allowed in our culture to dress wounds…” (In-depth Interview*, *a 48 year old male BU patient)*

According to participants, there were some few individuals who neither practiced witchcraft nor juju /charms but they naturally have evil eyes from birth. Such people have ‘bad luck’ so when they see your wound it will not heal fast. According to them this was one of the reasons why many people will not come to the clinic for treatment.

A 57 year old buruli ulcer patient made the following remark:

*‘‘…Even at the hospital/clinic*, *there are good hands and bad hands*. *Some nurses dress your wound and you will be relieved and not feel any pain until 3 days…*..*But some nurses also dress your wound and you will have no rest… It will pain you for the whole day or more…*. *When such people are dressing your wound*, *it does not heal fast*……*I would have loved that one nurse dress a wound for a patient till the wound gets healed because since I have been coming here for a long time*, *I know the nurses who have good hands and bad hands……*.*If I have my way*, *I would have maintained one nurse to treat my wound till it heals…*. *This may apply to many patients who come to the clinic for wound dressing because some see some nurses as having painful hands and some having good hands…*..*Some of the nurses too do not do their work with clear/free conscience so it affects the wound of the patients…*. *Such nurses do not help the wound to heal fast……But there are some nurses too who have clear/good conscience and are happy about what they do*. *Such nurses help the wound to heal fast…” (*A57 *year* old Buruli ulcer patient, in-depth interview).

Asked what has to be done to improve nurse-patient relationship to enhance effective wound care, this was what a respondent had to say:

*‘‘…Since I started coming to the clinic*, *I have seen significant improvement but I think some of the nurses need to be advised to treat us all well…*. *A wound is a painful condition so our dear nurses need to be patient with us so that we can remain at the hospital till our wounds heal…*..*By so doing*, *some of us will not be tempted to add other things to the wounds in our homes…”(*A 48 year old male *BU patient*, In-depth interview).

## Discussion

Consistent with earlier findings, this study revealed that cultural practices and beliefs significantly affected patients' wound care [6, 7, and 9]. The management of wounds in the home or formal health facilities is dependent on community ideas of wound categorisations of ‘normal’ or ‘abnormal’, which is similar to what was reported by Winch et al., [10] in their malaria study in Tanzania, where some conditions were referred to as ‘‘out-of-the ordinary fevers” or ‘‘fevers which do not respond to hospital treatment” and so were regarded as best treated by the traditional practitioners. Many individuals integrate both African and Western viewpoints into their belief systems comfortably without any contradictions as reported by Rudick, [11]. It became evident from the findings that wound related cultural beliefs have influenced how buruli ulcer wounds were treated in the study area. Whether a wound is treated at home or by traditional healers prior to being seen at the hospital or a combination of modern and traditional treatments is dependent on the dominant causal belief appealed to by the patients and his/her significant others. It is worthwhile noting that despite healers’ often low level of education, professionals such as teachers, nurses and ministers of religion have been found to use their services [12].

It worth reporting that beliefs on causation of wounds, perceived seriousness of buruli ulcer infection, perceived effectiveness of medical treatment, fear of recurring infections, surgery and amputation constitute socio-cultural features of buruli ulcer that promote preference for traditional/herbal treatment, which then causes delayed in seeking medical treatment [13, 14, 15, 16, and 17]. In this regard Kargbo-labour [18], recommended the need for African countries to consider local aetiology, perceptions and beliefs which are interwoven into the socio-cultural milieu of the African in disease control programmes. Findings from this study revealed that most respondents gave a maximum of three month for all normal wounds to heal and any wound that did not heal within three months was labeled as abnormal. These cultural beliefs could be impediments to buruli ulcer early case detection, treatment and adherence in the endemic communities of Ghana and other African countries [8]. This should be of concern to public health and a conscious effort should be made to understand the social, economic and cultural aspect of Buruli ulcer disease and its management in the local communities to aid its control.

Equally important is the fact that most patients in this study started self-care or treatment at home when they noticed a nodule, boil, plaque or sustain any wound. This finding corroborated the earlier work by Grietens et al., [7] where they reported that the commonest way of dressing wounds at home was the use of hot water to clean the wound and later applied ampicillin mixed in palm kernel oil. Herbs were used if after one month there was no improvement by way of healing. The self-care by buruli ulcer patients could be explained with the understanding of the clinical manifestations (signs and symptoms) of buruli ulcer where it starts as a painless itchy nodule, plague or wound [1, 19–21]. Moreover, this follows medical logic of given ‘first aid’ at home. This could therefore be termed as a ‘normal folk’ medical practice that is ‘rationally’ attempted by most human beings with a physical malaise. It came to light that Traditional healers become the next point of care if the home treatment seemed not to be working for the person. This confirms what was reported from a study conducted in Ghana, which showed that in most rural communities like elsewhere in sub-Sahara Africa, traditional healers were more accessible to the general population than biomedical service providers [19]. It has also been stated that there was approximately one traditional healer for about 500 people while the ratio of doctor to population is 1:40,000 [22].

The belief that some wounds are caused by evil spirits and witches has influenced the mode of treatment in buruli ulcer endemic communities, which therefore give currency to some of the healing practices devised by traditional healers, including the attempt to ‘‘drive” out the evil spirits from the wounds of patients to aid recovery [8, 10]. From respondents’ point of view, chronic wounds regarded, in some cases, as ‘‘bewitched wounds” cannot be treated by biomedical health practitioners. These wounds must of necessity be treated by traditional healers (spiritualists or herbalist) and this confirmed the belief reported byAgbenorku et al., and Winch et al., [20 and 10] that certain types of wounds and fevers are better treated by traditional methods and even made worse by Western medicine. It must be made clear that, in Buruli ulcer endemic communities these mainly include osteomyelitis and chronic leg ulcers. In fact, some patients do not seek any care for chronic ulcers because they are convinced that they will not be healed by help from biomedical practitioners because they were either bewitched or cursed to have the wound [24]. This was further explained by a study in Cameroon that although beliefs could influence health seeking behaviours for buruli ulcers, more compelling factors could also act on patients’ treatment paths, indicating that the choice of treatment was not decided upon solely with consideration to disease aetiology [7]. Similarly factors such as the effectiveness of treatment, place of treatment, difficulties of symptom recognition and acceptability of treatment were all paramount in deciding on treatment option to adopt [7].

Some of the beliefs associated with wound care are so strong that the people believed that any violation could lead to serious consequences such as the non-healing of wounds. These have serious effects on patients’ treatment seeking behaviour and adherence to biomedical treatments as they fit into the perception that some categories of wounds are not for biomedical treatments. For instance, buruli ulcer patients who believe that it is bad for a pregnant nurse to dress their wounds are more likely to drop out of treatment if a pregnant woman attends to them at any point in the management of their wounds. However, in the case of BU patients (both men and women), it is not about the opposite sex but the biological condition such as pregnancy status and age of the female provider was critical in accepting to be treated by her. As a result of these perceptions some patients may resort to other actions like treating their wounds with all kinds of concoctions including urine, which might expose the wound to secondary infections [1]. There is therefore the need for intensification of education in endemic communities to provide adequate information that will help to address some of the strongly held beliefs on wound care for a changed behaviour. In this direction, Ackumey et al., [25] suggested that providing education and knowledge at the individual level was not sufficient in itself to promote a change in behaviour. Health education programmes should be conducted to integrate the entire community especially traditional leaders, community volunteers, traditional healers and former patients to serve as change agents to help in accepting biomedical wound care [8].

This study has established the relationship between health care providers and their patients at the study area as an important element in treatment outcome [23]. It has demonstrated that respect for patients is an important step in addressing their health conditions and failure to do that would be a health service delivery in futility [11]. Some patients interviewed felt that they were not being treated well by health care providers and this must be addressed, if we want to win the confidence of patients to continue to accept and use the services provided at health facilities, it is often said that the best advertisement for a service provider are satisfied clients. Evidence from follow-ups on few dropped out patients revealed that the main reason why they defaulted or dropped out of treatment was disrespect or mistreatment by healthcare providers. Moreover, most clinicians do not listen to the patients well, especially with regards to their social, cultural and religious beliefs [11]. This might result in misunderstanding of some actions of the patients by health providers and also some actions of the health care providers by patients. This brings to the fore the recommendation that sociocultural factors affecting help-seeking practices for Buruli ulcer disease must feature strongly on the research agenda of the World Health Organisation (WHO) so as to generate data to guide public health strategies for the treatment and control of buruli ulcer in endemic countries [26].

## Supporting Information

S1 Appendix Tools for data collection. (DOCX)
