# Supplementary material for: Cultural Understanding of Wounds, Buruli Ulcers and Their Management at the Obom Sub-district of the Ga South Municipality of the Greater Accra Region of Ghana
Source: PLoS Negl Trop Dis. 2016 Jul 20;10(7):e0004825. doi: 10.1371/journal.pntd.0004825 (PMC4954709; doi:10.1371/journal.pntd.0004825)
Supplement: S1 Appendix — (DOCX) [file pntd.0004825.s001.docx]

**Appendix 1**

**SOCIO-CULTURAL FACTORS ASSOCIATED WITH BURULI ULCER MANAGEMENT IN THE OBOM SUB-DISTRICT OF THE GA SOUTH MUNICIPALITY OF THE GREATER ACCRA REGION OF GHANA**

Interviewers’ and FGD Guide for community elders’ and BU patients

Community elders’ perception of wound care and wound management.

**Introduction**

I am ………………, a research assistant from the Noguchi memorial Institute for Medical research and a PhD Student at the School of Public Health, University of Ghana, Legon. As you are aware, we have been conducting various researches under the auspices of the Stop Buruli project. One of the key elements of the project is the introduction of social interventions to support case detection and keeping them in treatment. And also how to effectively manage the conditions at the biomedical health facilities to prevent infections and the quick healing of the wounds. At this point, we want to interview some stakeholders to understand their view on these social supports and how the wounds would be managed. Please, you have been selected as one of the key informants to be interviewed because we know that your view will be very valuable to us as we move forward in the implementation of the project.

Thanks, in advance for accepting to speak with us on BU today. All your responses will be kept confidential and unless you permit it, your name will not be linked with anything that you will say in the course of this interview.

**In-depth Interviewers Guide**

1. Name
2. Sex
3. Age
4. Occupation
5. Ethnic background.
6. Have you managed a wound before?
7. Describe how wounds are managed from your community.
8. Do you know any herb which is used to dress wounds?
9. What times does one go to get the herbs?
10. Can anyone go for herbs to dress wounds?
11. Which types of people are qualified to manage/dress wounds?
12. Why should those people qualify to dress wounds?
13. Which people are not supposed to dress/manage wounds?
14. Why should those people not dress wounds?
15. Should those people dress wounds how will it affect the patients?
16. How will it affect them?
17. Has there been any evidence to show that certain people should not be allowed to dress wounds.
18. Have you ever met any of such people dressing wounds at the clinic?
19. What implication would that have on your wound?
20. Do you belief that the healing or none healing of wounds depend on cultural and religious factors?

**In-depth interviewers for BU patients.**

1. How did your condition start? Probe for the signs and symptoms.
2. What form of treatment did you resort to?
3. How were you being treated and how did you see the condition?
4. How long did you treat the condition with the form of treatment you were using?
5. What did you do after some periods?
6. How often were you dressing your wound in a day?
7. What kinds of drugs/medicines were used to dress your wounds? (Describe the process)
8. Mentioned the kinds of drugs/herbs used to dress your wound.
9. Was there any improvement in your condition?
10. Do you belief that some wounds are caused by witches/spirits? Prove how the beliefs affect treatment.
11. Do you have any belief that prohibits people from dressing wounds?
12. Who dressed your wound for you in the house?
13. Do you still use the herbs/drugs in addition to what is being given to you at the clinics?
14. Which kinds of people treat/dress your wounds at the clinic? Prove whether they are happy with the treatment.
15. Has any of the people mentioned in the question 11 dressed/treated your wound at the clinic? (if yes, prove whether they were happy with that)
16. Have you seen any improvement since you started coming for treatment at the clinic? Prove what needs to be done to improve wound dressing and treatment seeking among patients)

**SOCIO-CULTURAL FACTORS ASSOCIATED WITH BURULI ULCER MANAGEMENT IN THE OBOM SUB-DISTRICT OF THE GA SOUTH MUNICIPALITY OF THE GREATER ACCRA REGION OF GHANA**

Health System’s perception of the effective management of Buruli ulcer wounds to prevent secondary infections Key Informant Interviewers’ Guide

**Introduction**

I am ………………, a research assistant from the Noguchi memorial Institute for Medical research, and a PhD Student at the School of Public Health, University of Ghana, Legon. As you are aware, we have been conducting various researches under the auspices of the Stop Buruli project. One of the key elements of the project is the introduction of social interventions to support case detection and keeping them in treatment. And also how to effectively manage the conditions at the biomedical health facilities to prevent infections and the quick healing of the wounds. At this point, we want to interview some stakeholders to understand their view on these social supports and how the wounds would be managed. Please, you have been selected as one of the key informants to be interviewed because we know that your view will be very valuable to us as we move forward in the implementation of the project.

Thanks, in advance for accepting to speak with us on BU today. All your responses will be kept confidential and unless you permit it, your name will not be linked with anything that you will say in the course of this interview.

**Instruction to interviewer: Record the following in the note book before the start of the interview: *Name of interviewee (optional); highest level of Education/Training; Job title/position; Name of District and Community where the interview takes place***.

1. In your opinion is BU a major problem in this district? Why do you consider it as a major problem?
2. How many BU cases on average are seen in your facility each day? Probe, Can you say that the situation is getting better or worse in the past two years?
3. The cases that come to your facility, how to they come? Probe, Do they come by themselves or they are referred? Who referred them?
4. Does the health system have a programme to actively search for BU cases in the catchment communities? Probe for how is done? Who are responsible for the case search and referrals? How is the case searching done in affected communities? What are the challenges that impede active case searching in communities? How do you think that this could be overcome?
5. Is there any programme like community outreach to educate and create awareness on BU in the affected communities? Probe for who is responsible for it and how it is done? What are the challenges that impede active case searching in communities? How do you think that this could be overcome?
6. Is BU treatment completely free of charge to the patients? Probe for what does the patient have to pay for from his/her pocket? Is the treatment cost covered by the National Health insurance? Who pay for the free component of the treatment if any? Do you always have stock of the free component of the treatment available? What are the challenges that affect BU treatment in general?
7. At what stage do the patients report at the health facility? Probe for all the stages/categories of the disease.
8. Do BU patients who are not on admission attend health facilities regularly for treatment once they start treatment? Probe for dropout rate, non-adherence rate
9. Is your outfit aware of the any social intervention programme being implemented at the Obom health centre? Probe, could you please tell me what these interventions are?
10. Do you think that these interventions are necessary? How are they helping in terms of case searching and referral to the health centre, treatment dropout and adherence? What do you like about the interventions? What do you not like about it?
11. How is the intervention affecting BU treatment service delivery at the facilities? Probe, do you think that is affecting the facility positively or negatively, explain your position.
12. How are the BU patients treated at the health facility? Probe for the kind of treatment given at every stage.
13. Who dresses the wounds for the patients? Probe for how the ulcers are managed and the required times they are to be dressed a day/a week.
14. Do you think that the health system through the health service is providing the right medicines for the treatment of the conditions? Probe for how patients react to the treatment and the medicines.
15. Do the patients take care of the wounds well after dressing? Probe for the introduction of other herbs in the house after wound dressing.
16. Are you aware of any beliefs associated with the dressing/management of wounds in these communities? Probe for socio-cultural beliefs and dressing of wounds.
17. What other mechanisms do you think could be put in place to ensure early case detection, referral and better management of BU patients’ conditions at health facilities for treatment?
18. What are some of the challenges the health facilities are facing with the treatment/management of Buruli Ulcer in the district?
19. What are some of the challenges associated with BU patients and the management of their ulcers at the health centre? Probe for implications of home treatment, traditional medicine and cultural beliefs of community members.
20. If you were the Director General of Ghana health service, would you advocate for collaboration of biomedical treatment with traditional treatment? Probe for the reasons for and against the collaboration.
